# An expanded view of cell competition

**DOI:** 10.1242/dev.204212

**Published:** 2024-11-19

**Authors:** Ameya Khandekar, Stephanie J. Ellis

**Affiliations:** ^1^ Max Perutz Labs, Vienna Biocenter Campus (VBC), Dr.-Bohr-Gasse 9/Vienna Biocenter 5, 1030, Vienna, Austria; ^2^ University of Vienna, Center for Molecular Biology, Department of Microbiology, Immunobiology & Genetics, Dr.-Bohr-Gasse 9, 1030, Vienna, Austria; ^3^ Vienna BioCenter PhD Program, Doctoral School of the University of Vienna and Medical University of Vienna, A-1030, Vienna, Austria

**Keywords:** Cell competition, Cellular fitness sensing, Heterogeneity, Tissue robustness, Cell selection, Complex tissue organization

## Abstract

Cell competition arises in heterogeneous tissues when neighbouring cells sense their relative fitness and undergo selection. It has been a challenge to define contexts in which cell competition is a physiologically relevant phenomenon and to understand the cellular features that underlie fitness and fitness sensing. Drawing on examples across a range of contexts and length scales, we illuminate molecular and cellular features that could underlie fitness in diverse tissue types and processes to promote and reinforce long-term maintenance of tissue function. We propose that by broadening the scope of how fitness is defined and the circumstances in which cell competition can occur, the field can unlock the potential of cell competition as a lens through which heterogeneity and its role in the fundamental principles of complex tissue organisation can be understood.

## Teaser

**Summary:** A survey of cellular selection processes across diverse physiological contexts suggests that a broader view of the cell competition concept would benefit understanding of cell–cell interactions in heterogeneous tissues.

## Introduction

Cell competition is a conserved quality-control mechanism that serves to optimise the form and function of multicellular systems. In essence, it is considered to be a context-dependent, cell-elimination mechanism, mediated via short range cell–cell interactions, wherein fitter cells eliminate their less-fit neighbours (Baker, 2020; van Neerven and Vermeulen, 2023). Although the cell competition concept has gained traction, it has largely been restricted to describing competitive interactions in somewhat artificial experimental systems, wherein loser cell fate is mosaically induced in otherwise seemingly homogeneous tissues.

In reality, most tissues are highly heterogeneous, comprising complex micro-environments with multiple cell types and interactions, which are also influenced by systemic cues. Heterogeneity can occur at any scale within the organism, arising as a result of numerous intrinsic and/or extrinsic factors. We propose that the features underlying heterogeneity may have distinct impacts on fitness, and ultimately lead to specific, context-dependent mechanisms of cell selection. These salient features include the extent of heterogeneity, sensing mechanisms, context dependence and time scales (Box 1).

**Box 1. The nature of heterogeneity: impact on fitness and cell selection mechanisms**

Although all biological systems are heterogeneous, the nature of this heterogeneity could have variable effects on cellular fitness and, consequently, on cell selection mechanisms that are employed in a specific biological context (Table 1).

**Which features could describe and define heterogeneity, and have an impact on fitness?**

- Extent of heterogeneity
- Sensing mechanisms
- Context dependence
- Time scales

Despite any contention about what does – or does not – constitute cell competition, the one aspect that is generally agreed on is that it is a context-dependent phenomenon that depends on the relative phenotypic differences between neighbouring cells. This means that if there is no heterogeneity, there can be no cell competition and, thus, no means to adjust cellular composition of tissues as may be required. However, if heterogeneity is too extensive, then it becomes a challenge for cells to act collectively and maintain homeostasis. In line with this, many of the examples we discuss illustrate cases in which cell competition occurs to modulate the extent of heterogeneity.

We postulate that, especially in adult tissues, some degree of heterogeneity can be protective and facilitate more robust responses to unpredictable, highly variable challenges that may be encountered over long lifetimes. If this is true, biological systems need to have mechanisms in place that can help them sense how heterogeneous they are. How heterogeneity (and fitness) are sensed is an open question in the field. The existence of such mechanisms could dictate how permissive a system is to heterogeneity.

It is important to note that functional outcomes would be impacted by the time scales in which heterogeneity arises, how long it persists, and how fast selection programmes operate. Heterogeneity could be transient due to short-term fluctuations. In this scenario, it would only contribute towards shaping populations within a single generation. Conversely, transient fluctuations could cause permanent, inheritable changes to lineages. With changing biological contexts, distinct competitive advantages may be required by cells at different time scales, e.g. acute responses within the time span of a single cell cycle versus long-term selection across generations.

Taken together, a better understanding of heterogeneity will enable the field to uncover how fitness and cell selection mechanisms shape complex tissues and drive fitness across scales.

**Table 1.**
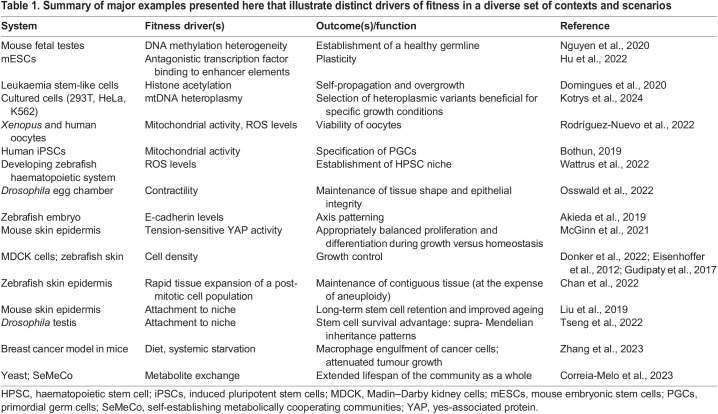
Summary of major examples presented here that illustrate distinct drivers of fitness in a diverse set of contexts and scenarios

**Fig. 1.**
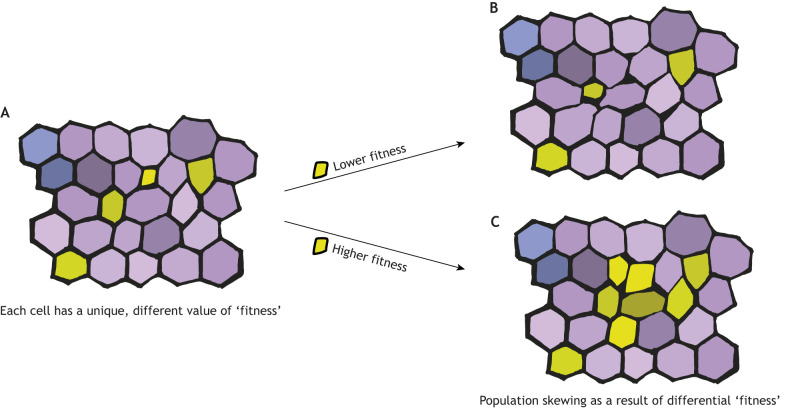
**Cellular heterogeneity and its possible impact on tissue-wide population distributions.** (A) Each cell has a unique ‘fitness value’ that differs from the fitness value of its neighbours. The difference in fitness values between cells is depicted by the degree of difference in their colours. (B,C) In specific contexts, some fitness values may be favoured over others, resulting in population skewing.

Although the functional consequences of heterogeneity across scales remain poorly understood, it is intuitive to expect that it results in cells possessing differing levels of ‘competitiveness’, i.e. the ability to persist within the population (Fig. 1A). Depending on the biological context, selection pressures may alter tissue composition and ultimately affect function (Fig. 1B,C). This makes it tempting to speculate that cellular selection processes – driven by cell competition and other related mechanisms – could be a pervasive and ever-present phenomenon to optimise organismal form and function, especially over long lifetimes.

How cell competition manifests in complex environments, and what functional consequences this has for tissue and organismal health have not been thoroughly investigated. Until recently, we have lacked the state-of-the-art technologies that could integrate cellular behaviours with systemic changes. Using integrative approaches and incorporating modern techniques such as single-cell-omics, spatial transcriptomics and advancements in microscopy, it is now possible to begin to tackle these complexities and bridge the scales between cellular and organismal level phenomena.

Another significant hurdle for cell competition research to overcome is the semantics and terminology that are used in the field. The challenge to define what constitutes the ‘fitness’ of a cell is the most glaring example of this problem (Box 2). There is no clear consensus on a definition of fitness, even in the field of evolutionary biology, from which the term is borrowed (Mobley, 2019). Historically, the most widely used definitions have been centred around growth and proliferation, i.e. reproductive fitness. Cell competition was first described in growing tissues wherein the main function of cells is to divide and grow (Morata and Ripoll, 1975; Simpson, 1979; Simpson and Morata, 1981); in this context, measuring fitness in terms of growth/survival is reasonable, but, nevertheless, such a singular definition imposes limits on the scope of how the concept may be applied to other functional states. Although newer studies are beginning to expand this perspective (Maheden et al., 2022), a growth-centric view of cell competition has hindered the inclusion of phenomena for which fitness is driven by other parameters, for example in tissues that have functions other than simply to grow, especially during homeostasis. It should be noted that the field of stem cell competition has emerged, which dissects the mechanisms by which stem cells compete for long-term occupancy of the adult stem cell niches: loser cells are evicted from the niche and undergo differentiation. However, the extent to which stem cell competition represents a subset of the cell competition concept has been a matter of some debate, in part because the fitness in stem cell competition is driven by stemness properties and not by proliferative capacity.

**Box 2. The challenge to define cellular ‘fitness’**

**Problems with defining ‘fitness’:**

- A complex, multi-trait concept; hard to define and measure.
- A frequently used term, but not always defined.
- Defined in several ways, leading to confusion or different interpretations of results.
- Current definitions centred around growth and survival; fail to include wider physiological phenomena.

**What could the salient features of cellular fitness be?**

- Is it an intrinsic or extrinsic property?
- Is it autonomous or non-autonomous?
- Is it heritable or stochastic?
- Is it dynamic or static?
- How is it sensed/detected?

**The need to expand the definition of fitness, where current definitions fail:**

- Cell fate decisions.
- Developmental programmes and outcomes.
- Complex interactions within a niche or a microenvironment.
- Tissues with specific functions.
- Maintenance of post-mitotic tissues.

**How can we improve the usage of the term ‘fitness’?**

1. Clearly define ‘fitness’ for every study.
2. Be more inclusive of fitness in diverse phenomena.
3. State the relationship between the definition and the measure of fitness.

In this Review, we describe ‘fitness’ to be a cellular feature driven by tissue functions inherent to specific physiological states. By viewing fitness differentials through the lens of cellular/tissue heterogeneity, we provide distinct examples from diverse tissues and contexts that could be considered to be examples of cell competition. Our examples cover fundamental cellular processes, including gene expression, metabolism, mechanics and cellular interactions with the microenvironment. We also use some of these examples to demonstrate how fitness-driven behaviours at the cellular scale are intertwined with those on a larger scale – at the tissue or systemic level. While the topics we have chosen for categorising our examples are vast fields unto themselves, here we highlight only certain aspects and discuss unique examples from them in the context of heterogeneity and fitness.

## Gene expression: harnessing inherently noisy processes to fine-tune tissue composition

Specification of cell fate is the functional output of gene expression programmes that are usually well-defined at the population level. However, at the level of single cells, considerable variability in gene expression – termed gene expression noise – exists and can have a bearing on precisely which individual cells within a tissue are specified to take on certain fates or not. Gene expression noise has long been considered a stochastic phenomenon. It is thought this stochasticity confers cells and organisms with evolutionary benefits, helping them adapt to fluctuating environments. During early development, intrinsic differences in gene expression in mouse embryonic stem cells (mESCs) influence developmental plasticity and robustness (Chakraborty et al., 2020; Martinez Arias and Brickman, 2011; Shahbazi et al., 2017). In microbial communities, heterogeneity in gene expression improves the fitness (i.e. the ability to produce progeny) of the community (Cerulus et al., 2016; Levy et al., 2012). Such mechanisms also operate within tumours, in which certain subpopulations of cancer cells may develop competitive fitness advantages over others, leading to clonal expansion, increasingly malignant phenotypes and poorer prognosis (Greaves and Maley, 2012; Salavaty et al., 2023; Yang et al., 2022). These examples illustrate that gene expression heterogeneity has deterministic outcomes and, therefore, can drive cellular fitness and, ultimately, cell competition. However, it has remained unclear if and how cells regulate this degree of stochasticity, either intrinsically or on a collective basis.

**Fig. 2.**
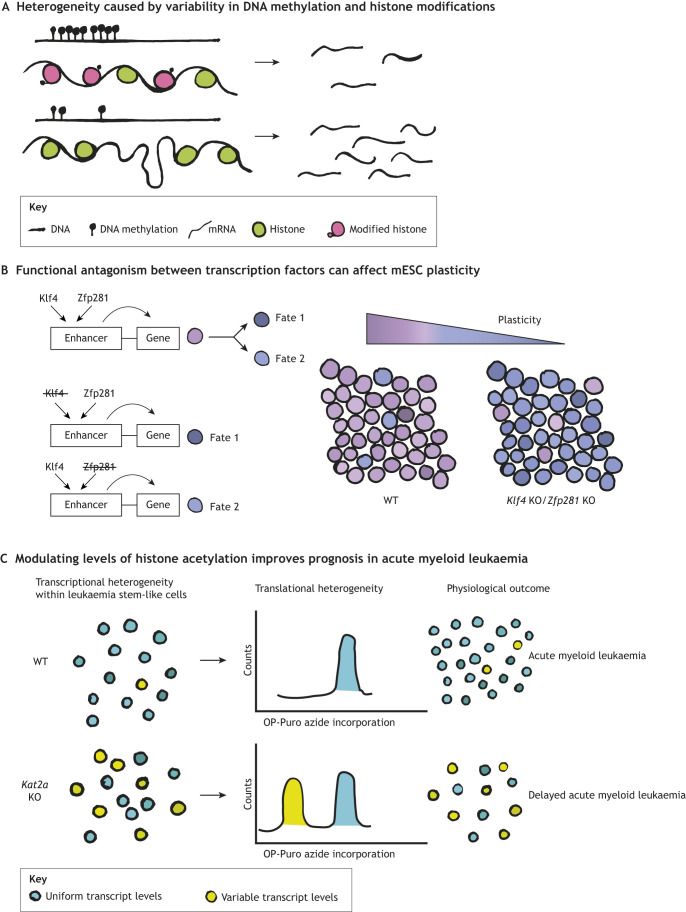
**Heterogeneity in gene regulation as a driver of cellular fitness.** Heterogeneity in factors regulating gene expression can have profound impacts on cellular functioning and, consequently, fate. (A) Epigenetic marks, such as DNA methylation, histone modifications and the binding of factors that read these marks, can affect transcription. (B) The antagonistic binding of transcription factors Klf4 and Zfp281 on differentially active transcribed enhancers (DATEs) confers plasticity, allowing for inter-convertibility of mouse embryonic stem cell (mESC) states. Loss of either *Klf4* or *Zfp281* leads to a decrease in plasticity and predisposes cells towards a particular fate. (C) Histone acetylation by Kat2a reduces transcriptional heterogeneity, which promotes the propagation of leukaemia stem-like cells in acute myeloid leukaemia. When *Kat2a* is knocked out in leukaemia stem-like cells, genes associated with ribosome assembly and translation have a decreased frequency of transcriptional bursting. A bimodal distribution seen in O-propargyl-puromycin (OP-Puro) incorporation assays in these knockouts suggests that loss of Kat2a leads to a decrease in translational activity of leukaemia stem-like cells, possibly leading to their depletion. KO, knockout; WT, wild type.

Gene expression is the culmination of a complex sequence of events that are subject to intrinsic and extrinsic factors. Aside from heterogeneities directly encoded in genomes by virtue of copy numbers, single-nucleotide polymorphisms (SNPs), etc. (Lee-Six et al., 2018, 2019; Martincorena et al., 2018, 2015; Moore et al., 2020), epigenetic marks such as DNA methylation also play a major role in modulating chromatin accessibility and the activities of gene regulatory elements and gene regulators (e.g. histone modifiers, transcription factors) (Fig. 2A). Heterogeneity in DNA methylation is only beginning to be examined (Lee et al., 2023; Lin et al., 2023; Xu et al., 2021) and has been reported in various scenarios, including during cell fate switching in mESCs (Singer et al., 2014; Smallwood et al., 2014), and a number of cancers potentially affecting clonal evolution and disease severity (Brocks et al., 2014; Sheffield et al., 2017; Wenger et al., 2019). Even subtle variations in DNA methylation can result in heritable, deterministic cell fate transitions, suggesting that heterogeneity in this process could act as a master regulator of cellular population dynamics. A striking example has been observed in the mouse fetal testes, where heterogeneity in DNA methylation underlies a selection programme that ultimately determines which germ cell precursors will differentiate and colonise the germline to make future gametes (Nguyen et al., 2020). Such germline selection programmes can foreseeably have consequences on gamete fitness in subsequent generations. While this example shows how fluctuations in DNA methylation can lead to long-term selection mechanisms, short-term fitness advantages may also arise in systems wherein methylation can be rapidly reversed (Zhang et al., 2020). The extent to which the observed heterogeneity is stochastic, versus shaped by active mechanisms, remains an important unanswered question.

Can the degree of stochasticity, and consequently the extent of heterogeneity, be controlled? While this remains an interesting avenue for further research, recent data suggest that cellular fitness can indeed be driven by modulating the degree of gene expression stochasticity. mESCs have emerged as an excellent system in which to study this question. mESCs inhabit distinct, and well-defined, gene expression states that mimic developmental transitions *in vivo*, ranging from highly pluripotent to primed for differentiation (Chakraborty et al., 2020; Filipczyk et al., 2015; Neagu et al., 2020; Shahbazi et al., 2017). The ability to switch reversibly between states confers plasticity and robustness, with gene expression heterogeneity biasing cell fate. Hu and colleagues recently identified a set of differentially active transcribed enhancers (DATEs) across interconverting mESC states (Hu et al., 2022). These enhancers preferentially regulate genes that are more variably expressed, having key roles in pluripotency and differentiation. The transcription factors Klf4 and Zfp281 compete to bind to DATEs and drive opposing transcriptional and chromatin programmes. Loss of this functional antagonism by knocking out either *Klf4* or *Zfp281* leads to a decrease in variation in gene expression. Functionally, this can be viewed as an effect on the ‘fitness’ of mESCs, by decreasing plasticity and influencing cell fate decisions on the population scale (Fig. 2B).

The example above shows how certain gene expression regulators increase cellular heterogeneity. However, the converse may also be true. Kat2a, a histone acetyl transferase, reduces transcriptional noise, decreasing heterogeneity in a mouse leukaemia stem cell model (Domingues et al., 2020). Conditional knockout of *Kat2a* reduces transcription factor binding and transcriptional burst frequency in a subset of gene promoters, thereby generating enhanced variability of transcript levels. This observation was subsequently harnessed to curtail the progression of acute myeloid leukaemia (AML) in the mouse model, which is characterised by abnormal progenitor self-renewal and defective white blood cell differentiation. Minimising heterogeneity favours the propagation of leukaemia stem cells; therefore, the authors knocked out *Kat2a* in leukaemia stem cells, which enhanced variability of transcription at certain loci and shifted the cell state from self-renewal to differentiation. This was likely due to the functional consequence of reduced translation in a subset of *Kat2a* knockout cells, which negatively impacted leukaemia stem cell maintenance/disease progression (Fig. 2C).

The cases described here indicate that ‘noise’ has biological meaning, and stochasticity can indeed be modulated. However, the extent to which this may be exploited for cellular selection programmes is still unclear. Recent technological advances in single-cell and spatial-omics are enabling us to explore deeper into heterogeneity within complex systems (Alon et al., 2021; Llora-Batlle et al., 2024 preprint; Oliveira et al., 2024 preprint). Further work that uncovers the molecular mechanisms underlying heterogeneity will shed light onto how complex multi-component systems harness it to drive biological outcomes.

## Mitochondrial function: a focal point for metabolic cellular selection programmes

It is well established that the metabolic status of the cell can have a deterministic outcome on cell fate and survival, and even drive cell competition (Banreti and Meier, 2020; Bowling et al., 2018; de la Cova et al., 2014; Lima et al., 2021; Pernaute et al., 2022; Zhang et al., 2023). Although this has been reviewed elsewhere (Esteban-Martínez and Torres, 2021; Lawlor et al., 2020; Nichols et al., 2022), here we highlight some recent examples that expand our view on the strategies that complex systems employ to optimise metabolic, and, consequently, cellular and tissue fitness in different scenarios.

**Fig. 3.**
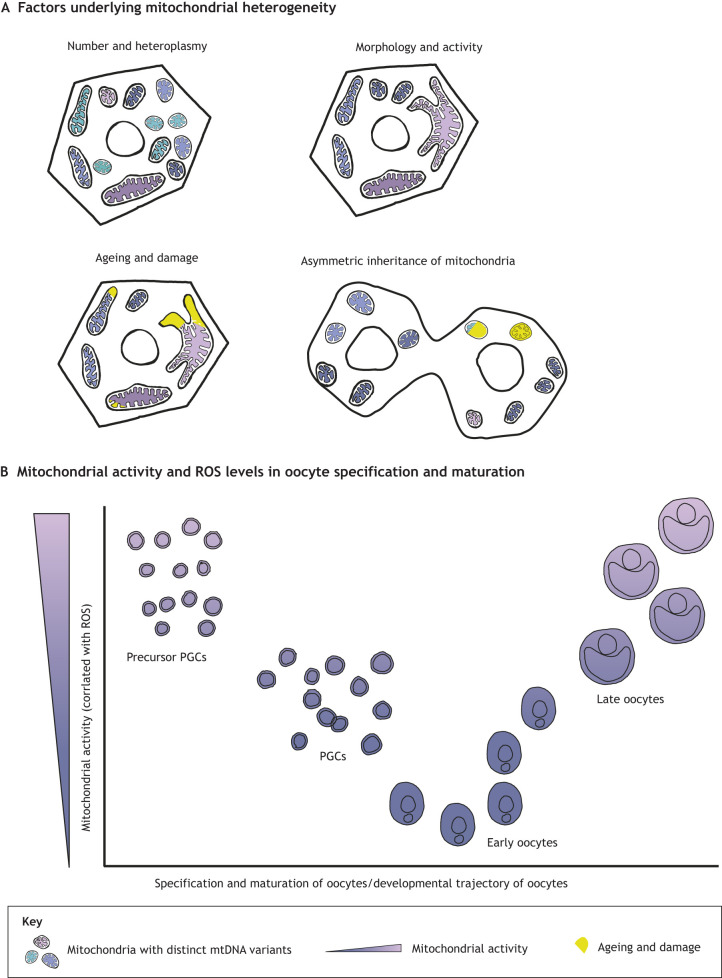
**Heterogeneity in mitochondrial form and function as a driver of fitness.** (A) Mitochondrial heterogeneity can manifest in many ways. This includes, but is not limited to, the number of mitochondria a cell may have, the degree of heteroplasmy within a cell or a population, the morphology and activity of mitochondria, and the degree of damage, which accumulates with age. Such heterogeneities may even arise due to asymmetric inheritance of mitochondria during cell division. (B) Functionally, such heterogeneities could have a profound impact on the metabolic status of, not just single cells, but across scales, even impacting traits at the level of the population. An example of this is offered by selection strategies amongst oocytes during their specification and maturation. Combining evidence from recent studies suggests that mitochondrial activity is a driver of this process. Primordial germ cells (PGCs) are specified *in vitro* by precursors that proliferate more slowly and have lower mitochondrial activity and, consequently, lower levels of reactive oxygen species (ROS). Early oocytes downregulate complex I of the mitochondrial electron transport chain, thereby lowering mitochondrial activity overall, allowing them to remain viable for extended periods. Restoration of complex I activity is associated with maturation into late oocytes. mtDNA, mitochondrial DNA.

Much like gene expression, the metabolic status of a cell is influenced by inherent heterogeneities present at multiple levels, which tend to accumulate with age (Granath-Panelo and Kajimura, 2024). Heterogeneities have been observed in the number of mitochondria in a cell, the degree of heteroplasmy [the presence of multiple variants of mitochondrial DNA (mtDNA)], mitochondrial morphology (which can be correlated with activity), the degree of damage present, and even as a result of asymmetric inheritance of differentially aged mitochondria during cell division (Agarwala et al., 2024; Katajisto et al., 2015; Kuznetsov and Margreiter, 2009; Stewart and Chinnery, 2015) (Fig. 3A). However, understanding how fitness is influenced by such heterogeneities has been technologically challenging. A recent study has utilised a novel strategy to track mtDNA heteroplasmy and ancestry of single cells in an ultra-high throughput manner (Kotrys et al., 2024). This approach allowed the authors to test how biological contexts drive the selection of advantageous mtDNA variants and shape population heteroplasmy. Interestingly, high levels of truncating complex I mtDNA heteroplasmy accumulated in cells grown in conditions wherein loss of mitochondrial complex I was beneficial, indicating that function can indeed modulate heterogeneity at the population level and consequently drive fitness.

In addition to producing metabolites needed for growth, repair, and homeostasis, metabolic processes also generate reactive oxygen species (ROS) that may cause damage. The balance between these useful metabolites and harmful ROS is a crucial determinant of lifespan and, consequently, cellular fitness, as has even been hinted at in cell competition models (Kohashi et al., 2021). How metabolic balance is achieved and modulated in different contexts to optimise function is not well understood.

Oocytes offer a fascinating context in which to explore this problem because they are specified during embryogenesis and remain viable for many decades; however, little is known about how oocytes maintain viability and why their quality declines with age. Rodríguez-Nuevo and colleagues recently showed that oocytes maintain viability through modulation of mitochondrial activity (Rodríguez-Nuevo et al., 2022). Early oocytes downregulate complex I of the electron transport chain, which decreases the production of ROS, thereby increasing their lifespan. In line with this observation, another study recently used pluripotent stem cells to demonstrate that the mitochondrial activity of the precursors of primordial germ cells (PGCs) influences their differentiation: slower proliferating precursors that have less-active mitochondria preferentially differentiate into PGCs *in vitro* (Bothun, 2019). Rodríguez-Nuevo and colleagues also found that, as oocytes mature, they begin to restore complex I activity and accumulate ROS. Taken together, specification and maturation of the oocyte seems to be linked to its metabolic status (Fig. 3B). ‘Fitness’ in this case reflects the metabolic need of the current cellular state during this process. It remains largely unclear how oocytes are selected to undergo maturation for each ovulation cycle. Future studies to examine mitochondrial heterogeneity within oocytes at any given point might be an interesting avenue through which we could learn more about oocyte maturation, and shed light on longstanding questions pertaining to female fertility.

Beyond the oocyte, strategies to minimise ROS may represent a more general mechanism to determine fitness and influence tissue composition across diverse cell types and physiological scenarios, especially within long-lived progenitor or stem cells. Cellular selection based on ROS levels has been reported in the developing zebrafish haematopoietic system (Wattrus et al., 2022). Haematopoietic stem cells (HPSCs), when stressed with high ROS, begin to display the phagocytic ‘eat-me’ signal, Calreticulin, on their cell surface. This signal promotes interactions with macrophages, which scan the surface of HPSCs, leading to one of two observed outcomes depending on the extent of Calreticulin surface expression. HPSCs harbouring high ROS-induced calreticulin are engulfed by macrophages, thus preventing their damage-prone progeny from contributing to the adult stem cell pool. By contrast, HPSCs showing lower levels of ROS are instead physically pruned by the macrophages through removal of HPSC cytosolic material, presumably leading to improved health of the individual HPSC. HPSCs that have been subject to pruning are more likely to undergo cell division, and thus make a greater clonal contribution to the adult stem cell pool. Although the molecular mechanisms remain to be parsed, these interactions demonstrate a metabolic quality control mechanism to ensure that the healthiest HPSCs establish and sustain haematopoiesis for the entire life of the organism. Despite a clear demonstration of a cellular selection mechanism, the extent to which this example fits the classic definition of cell competition is up for debate because the context dependency of the pruning mechanism was not addressed. If ROS were uniformly high across all HPSCs, would all HPSCs be equally pruned? The development of better tools to detect and manipulate ROS and other metabolic readouts *in vivo* will provide answers to these fascinating questions not only in HSPCs but also in the ovary.

These examples highlight the difficulty in applying classic cell competition criteria since metabolic processes are intertwined with cell-intrinsic survival mechanisms. Nevertheless, they advance our understanding of how distinct biological needs drive specific selection programmes to optimise metabolic fitness. Further characterisation of mitochondrial heterogeneity, its underlying causes, and how it changes in different contexts, will lead to a better understanding of how cellular fitness and cell competition shape tissues and organisms.

## Mechanical fitness: balancing cellular forces with growth control to maintain tissue form and function

Establishment and maintenance of tissue organisation is crucial for organismal health, making the underlying cellular processes entailed in morphogenesis a rich basis for potential cellular selection programmes. In this section, we discuss how sensing and modulation of cellular contractility and tissue mechanics can impinge on other cellular processes to prevent crowding, ensure controlled growth and promote correct form.

Sorting of cells into divergent lineage compartments based on differential cell adhesion underlies pattern formation and provides a physical means to keep cells with distinct identities separate from one another (Dahmann and Basler, 1999). However, the morphogen gradients that provide the initial cue to segregate lineages can be noisy. Cell competition – mediated through differential expression levels of the cell–cell adhesion protein E-cadherin (also known as cadherin 1) – was recently demonstrated to act to correct errors in these gradients during zebrafish anterior-posterior axis formation by eliminating cells in which E-cadherin levels are ‘unfit’ for their positional identity (Akieda et al., 2019). Disruption of this elimination mechanism has severe consequences for embryogenesis, underscoring the notion that cell competition is fundamentally intertwined with the biophysical mechanisms that drive tissue patterning.

Bulk mechanical properties of a tissue, such as tension or stiffness, are modulated to establish and maintain the specific tissue shapes that support organ function. These bulk features emerge from the mechanical properties of single cells, which are largely determined by actomyosin-dependent contractility and intercellular adhesion. Dynamic regulation of these molecular processes allows cells within tissues to push and pull on their neighbours and thus generate forces required for topological change. However, during growth, the same machinery also provides a means to sense growth rates of neighbouring cells, allowing for overall uniform tissue growth across a population of cells with heterogeneous proliferation rates. This concept – known as mechanical cell competition – was first proposed via a theoretical framework that predicted that as cell density increases, neighbours would impose increasing pressure on one another. This in turn could activate mechanotransduction pathways in neighbouring cells to adjust growth by triggering elimination of cells in regions that become too crowded to maintain homeostasis (Shraiman, 2005). Mechanical cell competition was subsequently confirmed experimentally in the *Drosophila* notum (Levayer et al., 2016; Moreno et al., 2019).

What is the basis for cellular selection in this case? It could be argued that, consistent with the classic cell competition definition, mechanical cell competition ultimately selects on cellular proliferation rate and favours the survival of cells that proliferate more quickly. However, the reality is more nuanced because vulnerability of any given cell to ‘sense’ a faster proliferating neighbour depends on its susceptibility to compaction, which is ultimately determined by its mechanical properties relative to its neighbours. Cell competition models using mosaics of wild-type cells and cells mutant for the basolateral polarity protein Scribble have emerged as an interesting context to examine this problem. In particular, in Madin–Darby canine kidney (MDCK) cell co-cultures of wild-type cells and cells expressing a short hairpin RNA (shRNA) against *Scribble*, sh*Scribble* cells lose, not because of a proliferative disadvantage, but rather because they have altered mechanical properties that make them more susceptible to deformation. Thus, upon contact with wild-type counterparts, sh*Scribble* cells undergo compaction, which triggers their apoptosis and extrusion from the tissue (Chen et al., 2012; Igaki et al., 2006; Norman et al., 2012; Wagstaff et al., 2016).

What is the consequence of failure to eliminate mechanically unfit cells? If softer, more-compressible cells are not removed from the tissue, their increased persistence in the tissue would eventually impact the bulk mechanical properties of the entire tissue and could disrupt tissue morphology. In line with this prediction, when *Scribble* is knocked down across entire tissue compartments, tissue architecture is severely disrupted and contributes to tumour progression (Bilder et al., 2000; Brumby and Richardson, 2003; Zhan et al., 2008). Surprisingly, mechanical cell competition may also form a key part of the infection response because it was recently shown that cells infected with bacterial pathogens exhibit altered mechanical properties relative to their uninfected neighbours. Uninfected cells are stiffer, enabling them to surround and compress the softer infected cells, eventually pushing them out of the tissue and limiting further propagation of infection (Bastounis et al., 2021).

**Fig. 4.**
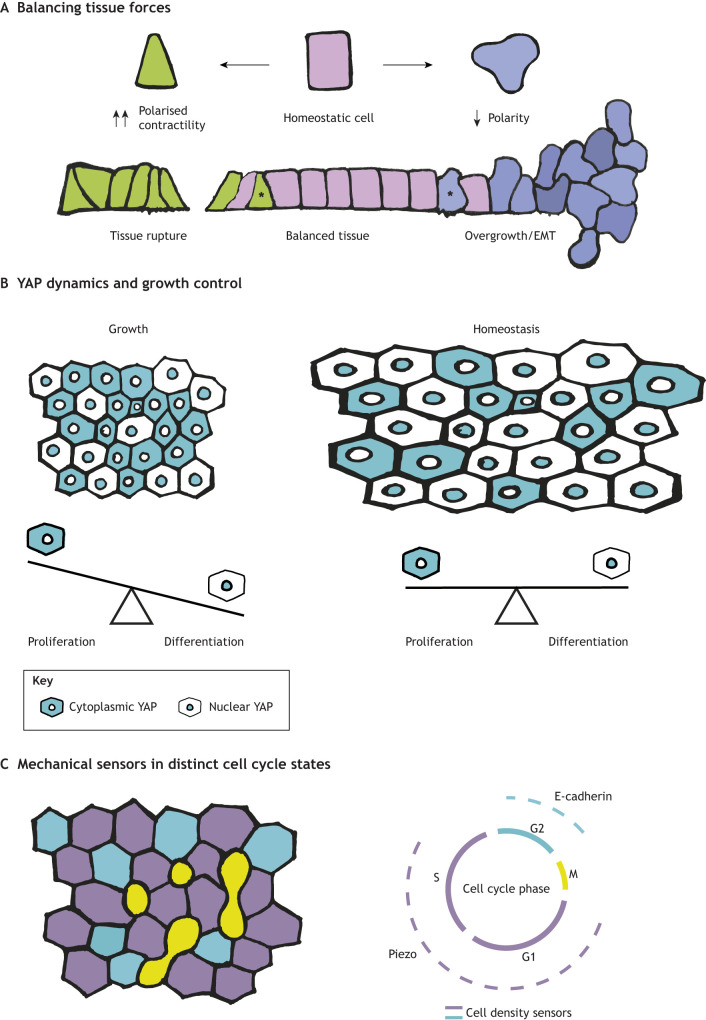
**Mechanical fitness to optimise form and function across scales.** (A) Forces between neighbouring cells and across the entire tissue must be balanced to preserve morphology and function. Disruption of mechanical properties (represented by the cells containing asterisks) may lead to the physical rupture of the tissue itself, or impact cell fate, leading to overgrowth, and in an epithelium, cause epithelial-to-mesenchymal transition (EMT). (B) Cell density can be sensed by molecules such as yes-associated protein (YAP). Dynamic shifts in YAP localisation can drive cell fate decisions and morphogenetic programmes. During growth, a majority of cells accumulate YAP in their cytosol, which favours self-renewal over differentiation. When tissue size plateaus, a greater proportion of cells have nuclear YAP, which promotes differentiation. The balance between these two states maintains homeostatic tissue size. (C) Similar mechanical sensors, Piezo1 and E-cadherin, could be involved in mechanical sensing in distinct cell-cycle states. Piezo largely exerts proliferative control of cells during G1 phase of the cell cycle, whereas E-cadherin does so at the G2-M transition.

The ongoing, unbalanced presence of cells that lie on the other end of the mechanical spectrum and are rather too stiff could also pose a challenge for maintaining tissue organisation. Recent optogenetic studies dissecting the role of the polarity component aPKC in the *Drosophila* egg chamber follicular epithelium showed that when aPKC is acutely disrupted, apical constriction is simultaneously increased in single cells, which increases tension across the entire sheet such that it is more vulnerable to rupture and epithelial tears; the same effect was also induced by acute induction of contractility by optogenetic RhoGEF2 activation (Osswald et al., 2022). Both manipulations also quickly disrupt overall tissue shape. This particular study underscores how cell-intrinsic mechanisms that control single-cell mechanics are crucial determinants of cellular fitness during homeostasis because they cumulatively impact bulk mechanical properties and overall tissue form. Moreover, forces must be balanced between neighbouring cells and across the entire tissue to ensure that tissue morphology, and thus function, can be maintained (Fig. 4A). Studies to better characterise contractile heterogeneity and probe the tissue-level mechanisms that coordinate force balance across homeostatic tissues to maintain organ function are an exciting avenue of future discovery.

Several molecular sensors of cell density have been identified that potentially play a role in maintaining tissue force balance. Hippo/yes-associated protein (YAP) signalling is one such candidate that could effectively be framed as a fitness sensor, and/or as a molecular determinant of fitness; relative differences in Hippo/YAP signalling have been previously shown to drive cell competition in many scenarios (Chen et al., 2012; Price et al., 2021; Suijkerbuijk et al., 2016; Ziosi et al., 2010). YAP is a co-transcriptional activator that translocates to the nucleus in response to increased cellular tension, where it induces TEAD-dependent gene expression. Thus, YAP provides a means to link mechanical stimuli to transcriptional responses and downstream fate decisions (Moya and Halder, 2019; Yu et al., 2015). Although the activity of YAP in single cells is heterogeneous, the collective pattern of YAP localisation across the entire tissue reflects its mechanical properties, which remain stable in homeostasis. However, in developing tissues, dynamic shifts in YAP localisation drive fate decisions and morphogenetic programmes (Brooun et al., 2022; Pan et al., 2018, 2016). This was recently exemplified in the growing basal epithelium of the mouse oesophagus (McGinn et al., 2021). During tissue growth, most basal cells harbour cytoplasmic YAP localisation, which favours self-renewal over differentiation, and thus drives net tissue expansion. However, as final tissue size is reached, the oesophageal epithelium is increasingly under tension, inducing nuclear accumulation of YAP in a greater proportion of cells in the population, thus pushing more cells towards differentiation. This finely tuned tensional homeostasis ensures that fate decisions in the epithelium are balanced and tissue size can be appropriately maintained (Fig. 4B). Active shifts in the population heterogeneity of YAP signalling impact fate decisions in other developing tissues as well, including the inner cell mass of mammalian blastocysts and during crypt morphogenesis in intestinal organoids (Hashimoto and Sasaki, 2019; Serra et al., 2019). Altogether, these observations imply that the heterogeneous patterns of YAP localisation observed in adult tissues must be tightly controlled, and selected for at the level of single cells, or else tissue form and function is at risk of disruption.

Other proteins have been identified that are similarly sensitive to cell density, including the mechanosensitive ion channel Piezo (Eisenhoffer et al., 2012; Gudipaty et al., 2017). However, heterogeneity of Piezo family proteins across tissues has yet to be investigated, leaving open the question of roles for Piezo1 in fitness and fitness sensing. The cell-cell adhesion protein E-cadherin has also been implicated as a sensor of cell density in MDCK cells. Intriguingly, Piezo largely impinges on proliferative control of cells during G1 phase of the cell cycle, whereas E-cadherin-dependent mechanosensing exerts control of the G2-to-M transition such that cells are restricted from entering mitosis at high density (Donker et al., 2022) (Fig. 4C). Together, this raises the possibility that there are distinct, cell cycle-dependent states of fitness and fitness sensing that collectively determine and sense mechanical fitness across solid tissues.

Coordination of cell-cycle phases between single cells within a tissue is a relatively understudied topic that may also underlie fitness. For example, cells poised in G2 could be more fit to respond to damage and quickly fill up space in the tissue. Could sensing of DNA content between cells be another crucial measure of cellular fitness that, together with mechanics, determines cellular dynamics? Such mechanisms would also provide a way to sense and eliminate genomically unstable, aneuploid cells that otherwise pose an oncogenic threat to tissue homeostasis, which is compatible with observations in flies (Ji et al., 2021). Curiously, in growing fish skin, terminally differentiated cells undergo a non-mitotic type of cell fission that allows rapid tissue expansion at the expense of maintaining complete karyotypes (Chan et al., 2022). This suggests that, in specific physiological contexts, the ‘normal’ modes through which proliferative fitness may be assessed do not apply, and that maintenance of tissue-level function at all costs could sometimes be the biggest selection pressure for single-cell survival.

The body of work we discuss here highlights the need for cross-disciplinary approaches that parse how growth and mechanics are integrated at the tissue level. Recently, spatial profiling was combined with computational pipelines to infer mechanical properties of cells and tissues *in situ* together with their transcriptional profiles (Hallou et al., 2023 preprint). Similarly spirited studies that harness and combine state-of-the-art technologies will illuminate fundamental cellular biophysical principles that govern which cells survive long term to support robust tissue function.

## Microenvironment: multi-scale, cross-tissue influences on cellular selection programmes

The immediate local milieu in which cells and tissues of multicellular organisms reside is known as the microenvironment. How cellular fitness and corresponding fitness-sensing mechanisms are impacted in different microenvironments is under-explored. This is in part because of a dearth of tractable experimental tools to tackle these questions, which bridge from the organismal to the molecular scale, and thus are not trivial to address. However, in recent years, emergent technologies from many fields have allowed us to begin to make inroads. Here, we discuss three intertwined aspects of microenvironment composition and how they may impact cellular fitness: (1) substrate and/or extracellular matrix (ECM) properties; (2) interactions between different cell types; and (3) the exchange of extracellular, systemic factors from the environment.

An emerging theme across stem cell systems is the role of cell–substrate interactions in dictating stem cell niche occupancy. In the mammalian inter-follicular epidermis, long-term niche residency and stem cell clonal dynamics are determined by expression of the hemi-desmosome protein COL17A1 (Liu et al., 2019). Progenitor clones with lower COL17A1 expression in the basal layer are more likely to divide asymmetrically and thus have a lesser contribution to the overall population. Rather counterintuitively, however, COL17A1 expression decreases with age, which is hard to reconcile if cells with high COL17A1 expression are being actively selected for. Nevertheless, it was recently reported that the density of the ECM component collagen underlies differences in epidermal clonal dynamics observed between ear skin and back skin, providing reinforcement to the notion that ECM properties impact stem cell fitness (Bansaccal et al., 2023). In the *Drosophila* male germline, a particularly interesting case that has inter-generational consequences for fitness has been described; germline stem cells mutant for the transcription factor Chinmo are transcriptionally rewired to express, not only more of the ECM component Perlecan (also known as Trol), but also the ECM receptors βPS-integrin and Dystroglycan (Tseng et al., 2022). This niche remodelling process allows *chinmo* mutant germline stem cells to outcompete wild-type stem cells for niche residency. Consequently, *chinmo* heterozygous mutants display non-Mendelian inheritance patterns, underscoring the multi-scale impact of mutations that impact on fitness in single cells, especially in the germline.

Cell–substrate interactions can also dictate the physical position of a cell in the niche, which is not a cell-intrinsic determinant of fitness but can greatly impact fate outcomes and long-term survival. This is beautifully demonstrated in the intestinal tract where stem cells reside in a niche known as the crypt, so-named for its invaginated, cup-like structure. Stem cells at the crypt base are more likely to self-renew; stem cells close to the edges of the crypt are more likely to migrate out of the crypt niche and terminally differentiate (Ritsma et al., 2014). In the large intestine, this organisation results in a spatial gradient of long-term stem cell potential that is purely determined by the position at which a stem cell is born in the crypt. Intriguingly, in the crypts of the small intestine, elegant live-imaging studies revealed that stem cells are endowed with cell mobility programmes that allow them to change their position; stem cells at the edge of the crypt niche can relocate to a more basal position, allowing them to improve their chances for long-term survival (Azkanaz et al., 2022). The molecular signals that govern this unique migratory behaviour are not yet understood, but it seems likely that stem cells that are primed to modulate cell–ECM adhesion and/or substrate attachment could effectively have an advantage over their neighbours, which allows them to traverse to the position in the niche that best supports long-term self-renewal.

Interactions with other cell types in a niche can also contribute to how fitness is measured. One basis of understanding for how this might work could come from the germinal centre reaction during B-lymphocyte development whereby interactions between B cells, T cells and dendritic cells lead to B-cell clonal selection. In this process, the B-cell clones with the highest affinity for antigen are selected for so they can mediate their subsequent function in humoral immunity (Victora and Nussenzweig, 2022). Somewhat analogously, within stem cell populations there is mounting evidence that interactions with stromal cells (Madan et al., 2019), and macrophages in particular, can influence clonal dynamics (Wattrus et al., 2022). Indeed, how tissue-resident immune cell populations shape competitive outcomes in the solid tissues they inhabit remains unexplored. Curiously, in the mouse epidermis, a transition in the mode of cell competition elimination mechanisms coincides with the colonisation of the epithelium by immune cells, raising the tantalising possibility that the two events are linked, and that the developing immune landscape influences fitness sensing and cell competition outcomes (Ellis et al., 2019). Further illustrating the physiological relevance of studying the impact of epithelial–immune cell interactions, recent data suggest that the competitive dynamics of growing cancer stem cells are also shaped by immune cells in the tumour microenvironment. In particular, a recent study examined a breast cancer model in which tumour growth is fuelled by upregulation of Myc and mTOR activity. This endows tumour cells with a ‘super competition’ phenotype – not only do they hyperproliferate, they also actively eliminate surrounding wild-type tissue to further enhance tumour growth (Zhang et al., 2023). However, under conditions of systemic starvation, tumour-associated macrophages undergo metabolic reprogramming that triggers them to engulf nearby cancer cells, attenuating competitive processes within the tumour, thereby slowing tumorigenesis.

**Fig. 5.**
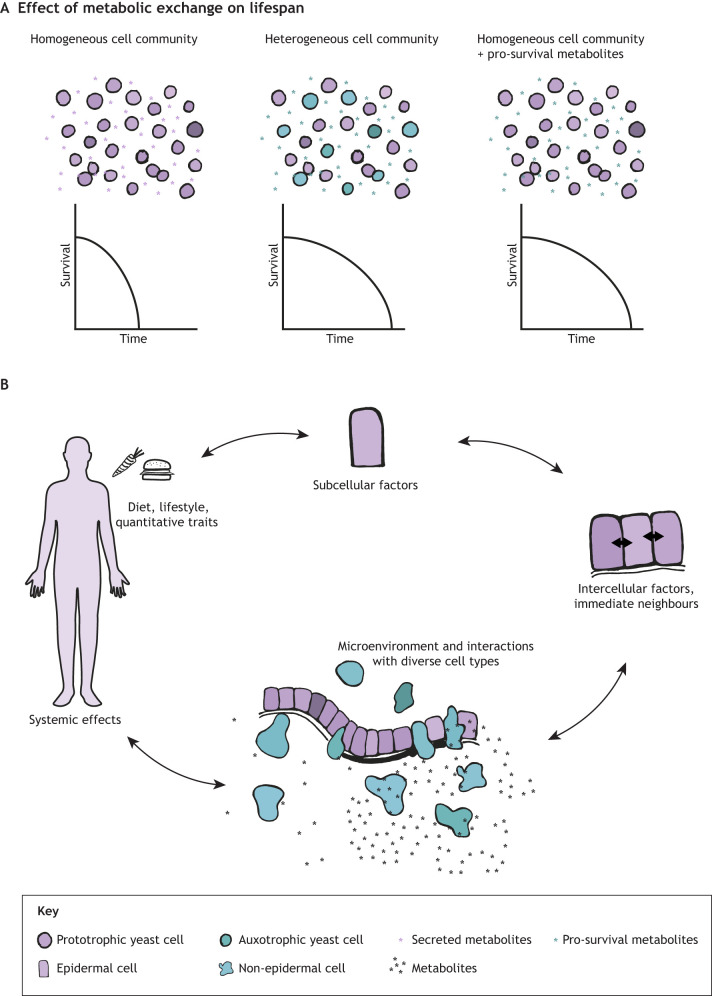
**The influence of the microenvironment on fitness and cell selection.** (A) Self-establishing metabolically cooperating communities (SeMeCo) of yeast cells show that metabolite exchange interactions in a heterogeneous community extend the lifespan of the community as a whole. Auxotrophic yeast cells (teal cells) produce pro-survival metabolites (teal asterisks) that lead to an extension of lifespan for the entire community. These pro-survival metabolites are sufficient for the extension of lifespan of homogeneous cellular communities as well. (B) Subcellular, cell-intrinsic factors that drive fitness are subject to factors across multiple scales, including interactions with immediate neighbours, the microenvironment and the niche, all of which are influenced by systemic inputs. The determination of fitness is a convergence of inputs from across all these scales, and optimises the form and function of the entire organism.

The prior example additionally highlights the relatively under-studied impact of systemic inputs, such as diet, on cellular fitness and fitness sensing in different physiological contexts. Tackling these mechanisms on the systemic level has been broached to some degree via dietary interventions (Herms et al., 2024; Sanaki et al., 2020; Sasaki et al., 2018), but it remains a challenge to parse specific effects following such global manipulations. Probing the impact of local nutrient or metabolite exchange between neighbouring cells and/or their more immediate microenvironment mitigates some of these challenges. An elegant example has recently been demonstrated to take place in heterogeneous communities of yeast (*Saccharomyces cerevisiae*) cells (Correia-Melo et al., 2023). Correia-Melo and colleagues show that metabolite exchange within a cellular community can extend the lifespan of the community as a whole. They used self-establishing, metabolically cooperating communities (SeMeCo) to test how metabolite exchange interactions in a heterogeneous community impact its lifespan. Interestingly, they noticed that cells unable to produce methionine imported it from the environment and rewired their metabolism to increase the export of protective metabolites. These protective metabolites were then imported by the methionine-producing cells, extending their lifespan, and consequently that of the entire community (Fig. 5A). This study provides a striking example of how genetic attributes of an individual cell within a population can modify the behaviour of neighbouring cells, and that this can have an impact on a larger scale. Metabolite exchange has also been reported to affect cell competition via lactate transfer from losers to winners in the Myc supercompetition model in the *Drosophila* wing disc (Banreti and Meier, 2020). Although progress is being made, significant further study and tool development is needed to improve our understanding of how diet, metabolites, or other extracellular factors can influence competitive cell interactions. The role of inflammation and/or infection in shaping fitness and fitness-sensing stand out as particularly intriguing unknown aspects, especially given the roles that immune signalling pathways (such as toll-like receptors, major histocompatibility complex proteins, and extracellular ATP) play in cell competition (Ayukawa et al., 2021; Germani et al., 2018; Meyer et al., 2014; Mori et al., 2022).

In summary, a mounting body of evidence illustrates the importance of the environment in dictating cellular fitness, strengthening the idea that individual cells operate as heterogeneous communities to influence biological function. Ongoing efforts to disentangle the distinct role of various niche components in determining tissue composition will push the frontier of our understanding not only of cell competition, but also more broadly of how cellular dynamics can be controlled in specific physiological states.

## Conclusion and discussion

Throughout this Review, we present examples of distinct putative cell competition mechanisms that operate on heterogeneous systems to establish and preserve robust tissue function. The studies we showcase demonstrate that heterogeneity, a key feature of life, can be flexibly harnessed to drive functional outcomes: selection of cells that are optimised for the necessary ‘fitness trait’. We propose to view ‘fitness’ as a feature that is driven by function, which is determined by the specific physiological state of the tissue, organ or even the entire organism. In light of this viewpoint, although many of the results we discuss fall outside of classical cell competition definitions, the outcome in every case is the same: cell selection for optimised tissue function.

Although a clear distinction between cell competition and cell selection exists (Baker, 2020; van Neerven and Vermeulen, 2023), we propose that the two phenomena ultimately converge on the same concept. Any differences are semantic, which, as per currently accepted definitions in the field of cell competition, lead to the exclusion of interesting examples of cell selection driving similar functional outcomes. Ultimately, both cell selection and cell competition serve to optimise the form and function of multicellular systems. The differences between the two processes largely lie in the mechanisms that they employ. Such mechanistic differences even exist among different cell competition models. For example, cell competition driven by copy number differences in ribosomal protein (minute) mutations and dMyc-driven super competition operate via distinct molecular mechanisms, but both lead to the elimination of loser cells from the *Drosophila* wing disc (Meyer et al., 2014). This underscores the notion that the field of cell competition would be strengthened by a broader, more inclusive definition.

A plethora of studies have directly demonstrated how competitive mechanisms can be co-opted by disease processes to drive progression (Alcolea et al., 2014; Flanagan et al., 2021; Krotenberg Garcia et al., 2021; van Neerven and Vermeulen, 2023; Zhang et al., 2023), or harnessed for tumour suppression (Brown et al., 2017; Colom et al., 2020, 2021; Gallini et al., 2023). Many more studies have indirectly hinted that cell competition mechanisms may even be a key factor in the highly clinically relevant issue of chemoresistance (Bacevic et al., 2017; Masud et al., 2022; Merino et al., 2019; Umkehrer et al., 2021). For example, there is intensive interest in how senescent cells, the presence of which in the tumour microenvironment often correlates with poor prognosis and therapy resistance, could be targeted by as-yet-unidentified fitness-sensing pathways to elicit their recognition as ‘loser’ cells and subsequent removal (D'Ambrosio and Gil, 2023; Huang et al., 2022; Nelson and Masel, 2017; Schmitt et al., 2022). Efforts to address this, and other crucial questions, will benefit enormously from input from cell competition experts who can provide an oft-neglected cell-biological perspective. Importantly, collaborative interactions will enable transfer of insights across disciplines from classical development systems to clinical applications.

We propose that by broadening the definition of ‘fitness’ to encompass more than just growth and proliferation, the concept of cell competition can gain wider traction (Box 2). A more inclusive view of cell competition will enable us to interrogate heterogeneities in complex multicellular systems, and how form and function can be optimised and fine-tuned in diverse biological contexts. Recent advances in spatial and -omics technologies have given us an excellent starting point to tackle such problems (Alon et al., 2021; Llora-Batlle et al., 2024 preprint; Oliveira et al., 2024 preprint). Further characterisation of inherent heterogeneity in various biological contexts, and the elucidation of mechanisms in distinct modes of cell competition will help us advance the field. The next technological bottleneck to be broached is the ability to integrate fate-tracking tools so that the functional consequences of spatial heterogeneity can be followed over time. Recently developed neighbourhood labelling strategies, such as uLIPSTIC or PUFFFIN, provide a historic record of cell–cell interactions between distinctly labelled cell populations (Lebek et al., 2024; Nakandakari-Higa et al., 2024). Foreseeably, such tools could be combined with barcoding and/or spatial-omics approaches to allow for better understanding of heterogeneity as local neighbourhoods evolve over time. Another powerful way to tackle these problems could be to design studies that transcend various scales, interrogating behaviour across the subcellular/cellular scale, the immediate cellular neighbourhood, the tissue/microenvironment and, lastly, in the context of the whole system (Fig. 5B). Integrating insights from across the different scales will provide a more complete picture of how heterogeneity influences tissue composition and organismal fitness across time. This will pave the way for a better understanding of the fundamental principles underling complex multicellular organisation in development, disease, ageing and evolution.
